# Elevated lipoprotein(a) and lipoprotein-associated phospholipase A_2_ are associated with unfavorable functional outcomes in patients with ischemic stroke

**DOI:** 10.1186/s12974-021-02359-w

**Published:** 2021-12-28

**Authors:** Xue Jiang, Jie Xu, Xiwa Hao, Jing Xue, Ke Li, Aoming Jin, Jinxi Lin, Xia Meng, Lemin Zheng, Yongjun Wang

**Affiliations:** 1 Beijing Tiantan Hospital, Capital Medical University, No. 119 South 4th Ring West Road, Fengtai District, Beijing, 100070 China; 2 China National Clinical Research Center for Neurological Diseases, Capital Medical University, Beijing, China; 3 Advanced Innovation Center for Human Brain Protection, Capital Medical University, Beijing, China; 4 The Institute of Cardiovascular Sciences and Institute of Systems Biomedicine, School of Basic Medical Sciences, Key Laboratory of Molecular Cardiovascular Sciences of Ministry of Education, NHC Key Laboratory of Cardiovascular Molecular Biology and Regulatory Peptides, Beijing Key Laboratory of Cardiovascular Receptors Research, Health Science Center, Peking University, No. 38 Xueyuan Road, Haidian District, Beijing, 100871 China; 5 Department of Neurology, Baotou Center Hospital, Inner Mongolia, China

**Keywords:** Lipoprotein(a), Lipoprotein-associated phospholipase A_2_, Inflammation, Functional outcomes, Ischemic stroke

## Abstract

**Background:**

The association of lipoprotein(a) [Lp(a)] and stroke functional outcomes was conflicting. The aim of the study was to clarify whether high Lp(a) is associated with unfavorable functional outcomes in patients with ischemic stroke.

**Methods:**

A total of 9709 individuals from the third China National Stroke Registry cohort were recruited. Plasma level of Lp(a) at admission was measured with enzyme-linked immunosorbent assay. The cut-off was set at the median for Lp(a). Functional outcome was assessed using the modified Rankin scale (mRS) at 3 months and 1 year after ischemic stroke. The association between Lp(a) and functional outcomes was evaluated using a logistic regression model.

**Results:**

The median age was 63.0 years, and 31.1% participants were women. Patients in higher Lp(a) group had higher incidences of unfavorable functional outcomes at 3 months. In logistic regression model, elevated Lp(a) levels were associated with unfavorable functional outcomes at 3 months (Q4 vs. Q1: odds ratio 1.33, 95% confidence interval 1.11–1.61). Subgroup analysis showed that in the lower Lp-PLA_2_ group, Lp(a) level was not associated with functional outcomes, but in the higher Lp-PLA_2_ group, Lp(a) level was significantly associated with functional outcomes. After grouped by different levels of Lp(a) and Lp-PLA_2_, the Lp(a) high/ Lp-PLA_2_ high group showed the highest incidence of unfavorable functional outcomes at 3 months and 1 year.

**Conclusions:**

Elevated Lp(a) level is associated with unfavorable functional outcomes in patients with ischemic stroke. The increment in both Lp(a) and Lp-PLA_2_ are associated with unfavorable functional outcomes at 3 months and 1 year after ischemic stroke.

**Supplementary Information:**

The online version contains supplementary material available at 10.1186/s12974-021-02359-w.

## Background

Lipoprotein(a) [Lp(a)] is composed of low-density lipoprotein (LDL)-like particle and apolipoprotein B-100 (apoB), which is linked to apolipoprotein(a) [apo(a)] by disulfide bond. The pathogenic characteristics of Lp(a) include proinflammatory, proatherogenic, and prothrombotic. The pro-inflammatory of Lp(a) is partially mediated by oxidized phospholipids attached to apo(a) [1]. Lp(a) has attracted considerable attention because of its several large clinical genetic observation studies, which confirmed that plasma Lp(a) level is positively associated with increased risks of stroke, [2] myocardial infarction [3, 4], and aortic valve stenosis [1, 5, 6].

Inflammation is involved in the occurrence and development of unfavorable functional outcomes in patients with ischemic stroke [7]. Lipoprotein-associated phospholipase A_2_ [Lp-PLA_2_], an inflammatory marker, is an independent predictor of ischemic stroke and coronary heart disease [8, 9]. More importantly, Lp-PLA_2_ is intimately associated with Lp(a) in atherosclerosis and cardiovascular disease [10]. Although previous studies examined the association of Lp(a) with risk of unfavorable functional outcomes in patients with ischemic stroke [11–13], rare studies have conducted the association of Lp(a) and Lp-PLA_2_ levels to stroke functional outcomes.

In this study, we aimed to evaluate the hypothesis that a high level of Lp(a) is associated with unfavorable functional outcomes and Lp(a) high/Lp-PLA_2_ high have a significant association with unfavorable functional outcomes in patients with ischemic stroke from the third China National Stroke Registry (CNSR-III) database.

## Methods

### Study population

We used the CNSR-III, a nationwide, prospective, multicenter, observational registration study based on etiology, imaging, and biology markers from patients with ischemic stroke and transient ischemic attack (TIA) between August 2015 and March 2018 in China [14]. Specific information about the database has been described in detail in our previous studies. [14]. All patients were enrolled within 7 days after symptom onset. We included 9709 individuals with complete information on plasma Lp(a) measurements. According to the principles mentioned in the Declaration of Helsinki, the ethics committees of Beijing Tiantan Hospital and all other recruited participating centers approved the study protocol [15]. Written informed consent was obtained from all participants (or guardians of participants) in this study.

### Baseline data collection

An electronic data capture system by face-to-face interviews was used to collect CNSR-III clinical baseline data. The subsequent data were gathered from the registry database, including age, sex, body mass index (BMI), and smoking status; medical history of hypertension, diabetes mellitus, hyperlipidemia and transient ischemic attack (TIA); systolic blood pressure (SBP), fasting plasma glucose (FPG), low-density lipoprotein cholesterol (LDL-C), high-density lipoprotein cholesterol (HDL-C), triglyceride (TG), high-sensitivity C-reactive protein (hsCRP), and lipoprotein-associated phospholipase A_2_ [Lp-PLA_2_]; stroke subtypes, classified as large artery atherosclerosis (LAA), cardioembolism (CE), small artery occlusion (SAO), other determined cause, or undetermined cause according to the Trial of ORG 10172 in Acute Stroke Treatment (TOAST) criteria [16], and other determined cause and undetermined cause are defined as Others [17], discharge medication rate of Lipid-lowering drugs and antiplatelet drugs, and the National Institutes of Health Stroke Scale (NIHSS) score at admission.

### Functional outcomes of stroke evaluation

The severity of ischemic stroke was evaluated using the NIHSS score at admission. Functional outcome was assessed with the modified Rankin scale (mRS) at 3 months and 1 year after stroke separately. The mRS scale ranges from 0 to 6. An mRS score of 0 was defined as no residual stroke symptoms; 5, severe disability; and 6, death. The mRS score of 0 to 2 points was defined as a favorable functional outcome, and mRS score of 3 to 6 points was defined as a unfavorable functional outcome [11, 12].

### Laboratory analyses

Fasting blood specimens from 10,491 patients were collected using EDTA anticoagulation tubes within 1 day after admission and were centrifuged on-site within 2 h of collection to separate plasma for subsequent testing. Standard hospital assays were used on fresh plasma samples to measure plasma FPG, LDL-C, HDL-C, TG, hsCRP, and Lp-PLA_2_.

### Lp(a) measurement

Lp(a) ELISA (Mercodia, Uppsala, Sweden) detects human Lp(a) and in terms of isoforms, is size-independent in terms of the kringle IV type 2 domain. The Mercodia ELISA is a solid phase two-site enzyme immunoassay and include a 5-point calibrator. The coefficient of variation (CV) was 7%.

### Statistical analyses

For displaying the information of the Lp(a) and functional outcomes more detailed, the Lp(a) quartiles were used for baseline characteristics classification and comparison. Furthermore, the cut-off was set at the median for Lp(a). Data on basic characteristics were presented as medians (interquartile ranges) for continuous variables. Categorical variables were presented as percentages. Nonparametric Wilcoxon test was used for comparisons of continuous variables, and chi-square test was used for comparisons of categorical variables among multiple groups. The associations of Lp(a) with mRS at 3 months and 1 year were examined using a logistic regression model. We adjusted the potential confounders measured at baseline in the analysis. The model was adjusted for age, sex, BMI, diabetes mellitus, LDL-C, HDL-C, TG, Lp-PLA_2_, TOAST subtype, and NIHSS score at admission. The strength of the associations was demonstrated using odds ratios (ORs) with 95% confidence intervals (CIs). The sensitivity analysis was used to rule out the effects of recurrence on the association between the levels of Lp(a) and outcomes at 3 months. Two-sided *p* < 0.05 was considered to be statistically significant. The above statistical analyses were conducted using SAS version 9.4 (SAS Institute Inc., Cary, North Carolina).

## Results

In brief, a total of 15,166 consecutive patients from 201 sites were recruited, among which 93.3% with ischemic stroke (*n* = 14,146) and 6.7% with TIA (*n* = 1020), According to the inclusion criteria, 1020 TIA patients were excluded from 15,166 patients. A total of 10,491 ischemic stroke patients’ blood samples were collected and examined at the laboratory, of which 664 patients lack of Lp(a) data and 118 patients lack of functional outcomes were excluded, and a total of 9709 patients were included in the study (Additional file 1: Figure S1). In Additional file 1: Table S1, the comparison of the included and excluded patients is shown. Compared with the excluded patients, the included patients tended to be older and had higher Lp-PLA_2_, and higher discharge medication rates of Lipid-lowering drugs and antiplatelet drugs.

### Baseline characteristics

Table 1 shows the baseline characteristics of the 9709 individuals stratified by Quartiles of baseline plasma Lp(a) levels. With the increment in plasma Lp(a) levels, patients tended to be older and had lower levels of BMI, FPG and TG, a higher proportion of LAA, and higher LDL-C, HDL-C, hsCRP, and Lp-PLA_2_.

**Table 1:** Baseline clinical characteristics

Characteristics	All (*n* = 9709)	Lp(a) (mg/dL)				*p-*value
		Quartile1 (*n* = 2428)	Quartile2 (*n* = 2426)	Quartile3 (*n* = 2428)	Quartile4 (*n* = 2427)	
		(< 8.9)	(8.9, 18.1)	(18.1, 35.8)	(> 35.8)	
Demographic characteristics						
Age, years, median (IQR), years	63.0 (55.0, 70.0)	62.0 (53.0, 70.0)	63.0 (55.0, 71.0)	63.0 (55.0, 70.0)	63.0 (55.0, 70.0)	< 0.0001
Male, *n* (%)	6689 (68.9)	1701 (70.1)	1680 (69.2)	1664 (68.5)	1644 (67.7)	0.34
BMI (kg/m^2^), median (IQR)	24.5 (22.6, 26.6)	24.6 (22.9, 26.7)	24.5 (22.6, 26.6)	24.4 (22.6, 26.6)	24.2 (22.5, 26.3)	0.001
Current smoker, *n* (%)	3102 (31.9)	780 (32.1)	766 (31.6)	790 (32.5)	766 (31.6)	0.86
Medical history, *n* (%)						
Hypertension	6112 (63.0)	1536 (63.3)	1517 (62.5)	1535 (63.2)	1524 (62.8)	0.94
Diabetes mellitus	2340 (24.1)	621 (25.6)	587 (24.2)	561 (23.1)	571 (23.5)	0.2
Hyperlipidemia	778 (8.0)	182 (7.5)	190 (7.8)	192 (7.9)	214 (8.8)	0.37
TIA, n (%)	221 (2.3)	57 (2.3)	57 (2.3)	57 (2.3)	50 (2.1)	0.88
TOAST subtype, *n* (%)						< 0.0001
LAA	2488 (25.6)	569 (23.4)	591 (24.4)	619 (25.5)	709 (29.2)	
CE	639 (6.6)	175 (7.2)	173 (7.1)	150 (6.2)	141 (5.8)	
SAO	2162 (22.3)	536 (22.1)	562 (23.2)	584 (24.0)	480 (19.8)	
Others	4420 (45.5)	1148 (47.3)	1100 (45.3)	1075 (44.3)	1097 (45.2)	
TPA, *n* (%)						
Yes	908 (9.3)	219 (9.0)	234 (9.6)	221 (9.1)	234 (9.6)	0.8
No	8801 (90.6)	2209 (91.0)	2192 (90.3)	2207 (90.9)	2193 (90.4)	
Laboratory test						
SBP at admission (mmHg), median (IQR)	149.0(135.0, 165.0)	148.5(135.0, 163.5)	149.5(136.5, 165.0)	149.5(135.0, 165.0)	149.0(135.0, 165.0)	0.32
FPG (mM), median (IQR)	5.6 (4.9, 7.0)	5.7 (5.0, 7.2)	5.6 (4.9, 7.1)	5.5 (4.9, 6.9)	5.5 (4.9, 6.8)	0.02
Baseline LDL-C (mM), median (IQR)	2.3 (1.8, 3.0)	2.1 (1.5, 2.7)	2.3 (1.7, 2.9)	2.4 (1.9, 3.1)	2.6 (2.0, 3.3)	< 0.0001
Baseline HDL-C (mM), median (IQR)	1.1 (0.9, 1.3)	1.0 (0.9, 1.3)	1.1 (0.9, 1.3)	1.1 (0.9, 1.3)	1.1 (0.9, 1.3)	< 0.0001
Baseline TG (mM), median (IQR)	1.4 (1.0, 1.9)	1.5 (1.1, 2.1)	1.4 (1.1, 1.9)	1.4 (1.0, 1.8)	1.3 (1.0, 1.7)	< 0.0001
Baseline hsCRP (mg/L), median (IQR)	1.8 (0.8, 4.8)	1.6 (0.8, 3.7)	1.8 (0.8, 4.7)	2.0 (0.9, 5.0)	2.0 (0.9, 5.9)	< 0.0001
Baseline Lp-PLA_2_ (ng/mL), median (IQR)	175.6 (127.3, 226.4)	168.2 (119.4, 217.0)	169.5 (123.0, 218.2)	177.7 (132.3, 228.8)	186.6 (137.1, 238.2)	< 0.0001
Discharge medication, *n* (%)						
Lipid-lowering drugs	9040 (93.4)	2254 (93.0)	2262 (93.6)	2249 (92.9)	2275 (94.0)	0.34
Antiplatelet agents	8925 (92.2)	2248 (92.7)	2211 (91.5)	2237 (92.4)	2229 (92.1)	0.4

Continuous data are presented as median (interquartile range, IQR), and categorical variables are presented as %

Lp(a): lipoprotein(a); BMI: body mass index; LAA: large-artery atherosclerosis; CE: cardioembolism; SAO: small artery occlusion; TIA: transient ischemic attack; TPA: tissue plasminogen activator; SBP: systolic blood pressure; FPG: fasting plasma glucose; LDL-C: low-density lipoprotein cholesterol; HDL-C: high-density lipoprotein cholesterol; TG: triglyceride; hsCRP: high-sensitivity C-reactive protein; Lp-PLA_2_: lipoprotein-associated phospholipase A_2_

### Association between the levels of Lp(a) and functional outcomes at 3 months and 1 year

**Fig. 1:**
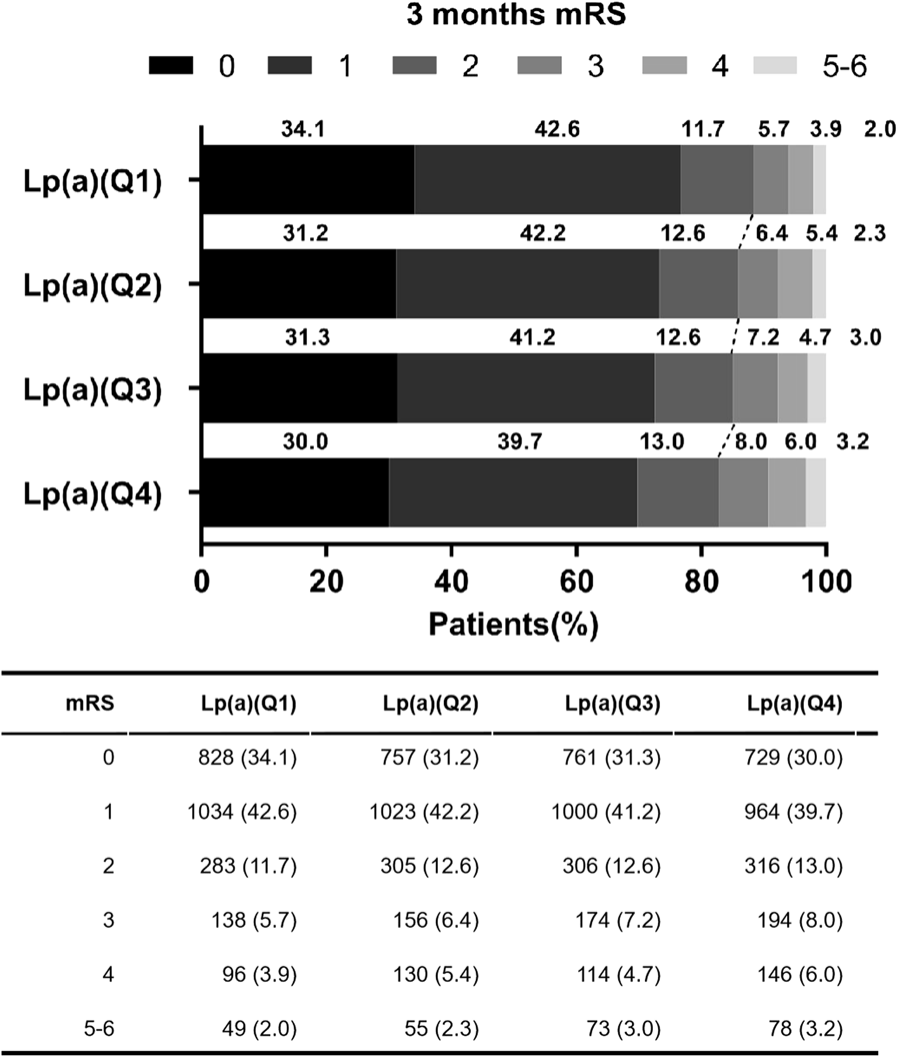
**A**, **B** Functional outcomes at 3 months based on mRS, presented as percentage

Additional file 1: Figure S2 demonstrates a positive association between the levels of Lp(a) and functional outcomes of stroke at 3 months. In the unadjusted model, elevated levels of Lp(a) were positively associated with the unfavorable functional outcomes of stroke as evaluated using mRS score ≥ 3 at 3 months [Quartile 4 vs. Quartile 1, OR 1.58, 95% CI 1.34–1.86, *p* < 0.0001] (Table 2). Furthermore, elevated Lp(a) levels were significantly associated with the unfavorable functional outcomes of stroke as evaluated by mRS score ≥ 3 at 1 year [Quartile 4 vs. Quartile 1, OR 1.46, 95% CI 1.23–1.72, *p* < 0.0001]. After adjustment for age, sex, BMI, diabetes mellitus, LDL-C, HDL-C, TG, Lp-PLA_2_, TOAST subtype, and NIHSS score at admission, similar results were observed. Elevated levels of Lp(a) were positively associated with the unfavorable functional outcomes of stroke as evaluated by mRS score ≥ 3 at 3 months [Quartile 4 vs. Quartile 1, OR 1.33, 95% CI 1.11–1.61, *p* < 0.0001] and at 1 year [Quartile 4 vs. Quartile 1, OR 1.25, 95% CI 1.04–1.51, *p* < 0.0001]. Distribution of mRS scores at 3 months according to Lp(a) levels showed similar trends (Fig. 1).

**Table 2:** Association between the levels of Lp(a) and functional outcomes at 3 months and 1 year

	Event rate	OR (95% confidence interval)	
		Unadjusted	Adjusted
mRS ≥ 3 at 3 months			
Lp(a)(Q1)	11.66	Reference	Reference
Lp(a)(Q2)	14.06	1.24 (1.05–1.47)	1.20 (0.99–1.45)
Lp(a)(Q3)	14.87	1.32 (1.12–1.56)	1.24 (1.03–1.50)
Lp(a)(Q4)	17.22	1.58 (1.34–1.86)	1.33 (1.11–1.61)
mRS ≥ 3 at 1 year			
Lp(a)(Q1)	11.52	Reference	Reference
Lp(a)(Q2)	13.36	1.19 (1.00–1.41)	1.12 (0.92–1.35)
Lp(a)(Q3)	14.01	1.25 (1.06–1.49)	1.18 (0.97–1.42)
Lp(a)(Q4)	15.95	1.46 (1.23–1.72)	1.25 (1.04–1.51)

Adjust for age, sex, BMI, Diabetes mellitus, LDL-C, HDL-C, TG, Lp-PLA_2_, TOAST subtypeand NIHSS score at admission

BMI: body mass index; LDL-C: low-density lipoprotein cholesterol; HDL-C: high-density lipoprotein cholesterol; TG: triglyceride; Lp-PLA_2_: lipoprotein-associated phospholipase A_2_; NIHSS: National Institutes of Health Stroke Scale

### Subgroup analysis for the association between Lp(a) levels and mRS ≥ 3 at 3 months and 1 year

**Fig. 2:**
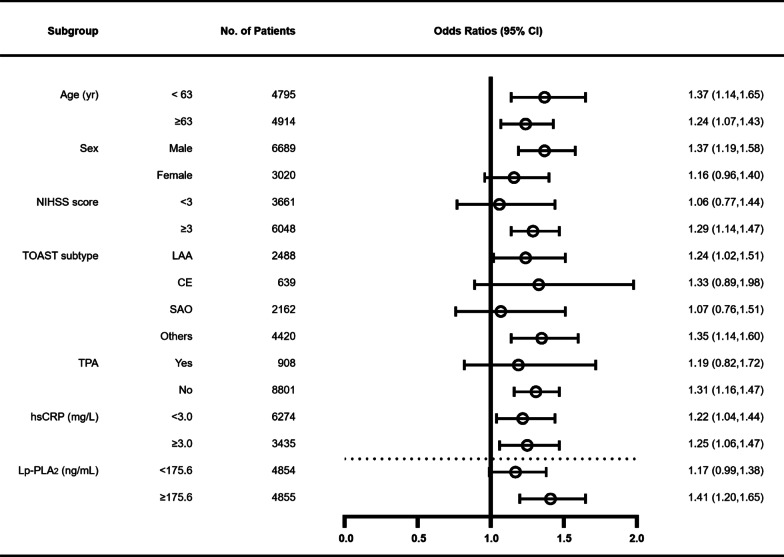
Forest plot of subgroup analysis for the association between Lp(a) levels and mRS ≥ 3 at 3 months. The cut-off of continuous variable was the median value except for hsCRP. NIHSS: National Institutes of Health Stroke Scale; LAA: large-artery atherosclerosis; CE: cardioembolism; SAO: small artery occlusion; TPA: tissue plasminogen activator; hsCRP: high-sensitivity C-reactive protein; Lp-PLA_2_: lipoprotein-associated phospholipase A_2_

Figure 2 shows that in the lower Lp-PLA_2_ group, Lp(a) level was not associated with functional outcomes, but in the higher Lp-PLA_2_ group, Lp(a) level was significantly associated with functional outcomes. We then used Lp-PLA_2_ for subgroup analysis to further evaluate the association between Lp(a) levels and functional outcomes of stroke assessed using mRS ≥ 3 at 3 months and 1 year.

### Association of functional outcomes grouped by different levels of Lp(a) and Lp-PLA_2_

Table 3 shows the association of functional outcomes grouped by different levels of Lp(a) and Lp-PLA_2_ (< median value vs. ≥ median value). Compared with Lp(a) low/ Lp-PLA_2_ low group, patients with Lp(a) high/ Lp-PLA_2_ high group showed a significant association with unfavorable functional outcomes at 3 months (OR 1.21, 95% CI 1.01–1.46) and 1 year (OR 1.25, 95% CI 1.03–1.51) after ischemic stroke.

**Table 3:** Association of functional outcomes grouped by different levels of Lp(a) and Lp-PLA_2_

	Event rate	OR (95% confidence interval)	
		Unadjusted	Adjusted
mRS ≥ 3 at 3 months			
Lp(a) < median and Lp-PLA_2_ < median	12.80	Reference	Reference
Lp(a) < median and Lp-PLA_2_ ≥ median	12.92	1.01 (0.85–1.17)	0.96 (0.79–1.17)
Lp(a) ≥ median and Lp-PLA_2_ < median	14.64	1.17 (0.99–1.38)	1.08 (0.90–1.30)
Lp(a) ≥ median and Lp-PLA_2_ ≥ median	17.28	1.42 (1.22–1.66)	1.21 (1.01–1.46)
mRS ≥ 3 at 1 year			
Lp(a) < median and Lp-PLA_2_ < median	12.11	Reference	Reference
Lp(a) < median and Lp-PLA_2_ ≥ median	12.82	1.07 (0.90–1.27)	1.08 (0.89–1.32)
Lp(a) ≥ median and Lp-PLA_2_ < median	14.26	1.21 (1.02–1.43)	1.14 (0.95–1.37)
Lp(a) ≥ median and Lp-PLA_2_ ≥ median	15.61	1.34 (1.14–1.58)	1.25 (1.03–1.51)

Adjust for age, sex, BMI, Diabetes mellitus, LDL-C, HDL-C, TG, TOAST subtypeand NIHSS score at admission

BMI: body mass index; LDL-C: low-density lipoprotein cholesterol; HDL-C: high-density lipoprotein cholesterol; TG: triglyceride; NIHSS: National Institutes of Health Stroke Scale

### Sensitivity analysis

#### Association between the levels of Lp(a) and functional outcomes at 3 months after removing patients with recurrent stroke

To rule out the effect of recurrence on the association between the levels of Lp(a) and functional outcomes at 3 months, we excluded the 3 months recurrent stroke population for further analysis. As shown in Additional file 1: Table S2, in the unadjusted model, elevated levels of Lp(a) were positively associated with the unfavorable functional outcomes of stroke as evaluated by mRS score ≥ 3 at 3 months. After adjustment for age, sex, BMI, diabetes mellitus, LDL-C, HDL-C, TG, Lp-PLA_2_, TOAST subtype, and NIHSS score at admission, similar results were observed. Elevated levels of Lp(a) were positively associated with the unfavorable functional outcomes of stroke as evaluated by mRS score ≥ 3 at 3 months.

## Discussion

In the CNSR-III cohort study, we investigated the association between plasma Lp(a) levels and the functional outcomes of ischemic stroke. The results demonstrated a positive association between the levels of Lp(a) and functional outcomes evaluated by mRS at 3 months and 1 year after stroke. The association remained after excluding patients with recurrence of stroke at 3 months. More importantly, compared with Lp(a) low/ Lp-PLA_2_ low group, patients with Lp(a) high/ Lp-PLA_2_ high group showed a significant association with unfavorable functional outcomes at 3 months and 1 year after ischemic stroke.

In recent years, several small clinical studies have demonstrated that elevated Lp(a) levels are positively associated with unfavorable functional outcomes in patients with ischemic stroke. In a clinical study by Wang et al. who included 232 consecutive patients with an acute ischemic stroke diagnosis complicated with type 2 diabetes, higher Lp(a) levels at admission are associated with increased risk of unfavorable functional outcomes at 3 months according to mRS scores [11]. Similarly, in a study conducted by Wang et al., who investigated 153 patients with acute ischemic stroke and 120 controls, an increased risk of unfavorable functional outcomes was associated with Lp(a) levels [12]. In another study that recruited 100 consecutive patients with acute ischemic stroke and 120 controls, a positive association is suggested between Lp(a) levels and poorer long-term prognosis of stroke [18]. By contrast, Kooten et al. failed to find any association of stroke prognosis with Lp(a) levels [13]. The association of stroke prognosis with Lp(a) level remains unclear. Therefore, a large sample cohort study is warranted to further clarify the association of ischemic stroke prognosis with Lp(a) levels. The current results demonstrated a positive association between the levels of Lp(a) and functional outcomes evaluated by mRS at 3 months and 1 year after stroke. To remove the effect of stroke recurrence on the conclusion, we further analyzed the association between Lp(a) levels and functional outcomes after excluding patients with stroke recurrence at 3 months, the association still existed. Our study confirmed a positive association between plasma Lp(a) levels and functional outcomes at 3 months and 1 year after ischemic stroke.

The mechanism through which Lp(a) levels are associated with functional outcomes of ischemic stroke remains unclear until now. Inflammation runs through the onset, process, and progression of acute ischemic stroke. In acute ischemic stroke, inflammatory cascade affects functional outcomes [7]. Our study revealed that the increment in both Lp(a) and Lp-PLA_2_ are associated with unfavorable functional outcomes at 3 months and 1 year after ischemic stroke. The pathogenic effect of Lp(a) is partly due to its pro-inflammatory effect, which is harmful to the progression of ischemic stroke. The pro-inflammatory effect including endothelial inflammation is mediated partially by its oxidized phospholipid (OxPL) content [19]. As a pro-inflammatory indicator, Lp-PLA_2_ is mainly expressed on the surface of inflammatory cells enriched in plaques, and it promotes the secretion of inflammatory mediators by degrading OxPL to cause endothelial dysfunction [20]. Endothelial dysfunction is associated with unfavorable functional outcomes of ischemic stroke [21, 22]. In addition, observational studies have shown plausible mechanisms through which Lp(a)–Lp-PLA_2_–OxPL may mediate atherosclerosis and cardiovascular disease [10, 23]. In summary, we speculate that Lp(a) may aggravates the inflammation mediated by Lp-PLA_2_, leading to endothelial dysfunction and unfavorable functional outcomes of ischemic stroke.

The present study is the largest by far to evaluate the clear association between plasma Lp(a) levels and functional outcomes after ischemic stroke. However, it has several limitations. First, we only measured Lp(a) at admission, and no serial measurement of Lp(a) levels was performed. For this reason, we could not conclude any causal relationship of functional outcomes with high Lp(a) levels. We only demonstrated a positive association of unfavorable functional outcomes after ischemic stroke with high Lp(a) levels. Second, the study samples comprised Chinese individuals, limiting the application of its conclusions to other races and populations. Third, genetic data were lacking because of the observational nature of the study. Future studies are warranted to further investigate the effect of these factors on Lp(a) and functional outcomes in patients with ischemic stroke.

## Conclusions

Elevated Lp(a) level is associated with unfavorable functional outcomes evaluated by mRS at 3 months and 1 year after ischemic stroke. The increment in both Lp(a) and Lp-PLA_2_ are associated with unfavorable functional outcomes at 3 months and 1 year after ischemic stroke. Further studies should be carried out regarding the inflammation mechanism between increased Lp(a) levels and unfavorable functional outcomes in patients with ischemic stroke.

## Supplementary Information

**Additional file 1.** Table S1. Comparisons of Baseline characteristics between included patients and excluded patients. Table S2. Association between the levels of Lp(a) and functional outcomes at 3 months after excluding stroke recurrence. Table S3. Association between the Lp-PLA2 and functional outcomes at 3 months and 1 year. Table S4. Association between the levels of Lp(a) and functional outcomes at 3 months and 1 year. Table S5. Comparisons of Baseline characteristics between included patients and excluded patients. Table S6. Association between the levels of Lp(a) and functional outcomes at 3 months and 1 year. Table S7. Association of functional outcomes grouped by different levels of Lp(a) and Lp-PLA2. Figure S1. Flowchart of patients’ inclusion analysis strategy. Figure S2. The association between the levels of Lp(a) and outcomes at 3 months. Figure S3. The association of Lp(a) and functional outcomes in patients with ischemic stroke at 3 months and 1 year.

## Data Availability

The data that support the findings of this study are available from the corresponding author upon reasonable request.

## Abbreviations

Lp(a): Lipoprotein(a)
CNSR-III: The third China National Stroke Registry
mRS: The modified Rankin scale
NIHSS: The National Institutes of Health Stroke Scale
TIA: Transient ischemic attack
BMI: Body mass index
TOAST: Trial of ORG 10172 in Acute Stroke Treatment
LAA: Large artery atherosclerosis
SAO: Small artery occlusion
CE: Cardioembolism
SBP: Systolic blood pressure
FPG: Fasting plasma glucose
LDL-C: Low-density lipoprotein cholesterol
HDL-C: High-density lipoprotein cholesterol
TG: Triglyceride
hsCRP: High-sensitivity C-reactive protein
Lp-PLA_2_: Lipoprotein-associated phospholipase A_2_
