# Supplementary material for: Elevated lipoprotein(a) and lipoprotein-associated phospholipase A2 are associated with unfavorable functional outcomes in patients with ischemic stroke
Source: J Neuroinflammation. 2021 Dec 28;18:307. doi: 10.1186/s12974-021-02359-w (PMC8715597; doi:10.1186/s12974-021-02359-w)
Supplement: Supplementary file 1 — Additional file 1. Table S1. Comparisons of Baseline characteristics between included patients and excluded patients. Table S2. Association between the levels of Lp(a) and functional outcomes at 3 months after excluding stroke recurrence. Table S3. Association between the Lp-PLA2 and functional outcomes at 3 months and 1 year. Table S4. Association between the levels of Lp(a) and functional outcomes at 3 months and 1 year. Table S5. Comparisons of Baseline characteristics between included patients and excluded patients. Table S6. Association between the levels of Lp(a) and functional outcomes at 3 months and 1 year. Table S7. Association of functional outcomes grouped by different levels of Lp(a) and Lp-PLA2. Figure S1. Flowchart of patients’ inclusion analysis strategy. Figure S2. The association between the levels of Lp(a) and outcomes at 3 months. Figure S3. The association of Lp(a) and functional outcomes in patients with ischemic stroke at 3 months and 1 year. [file 12974_2021_2359_MOESM1_ESM.doc]

**SUPPLEMENTAL MATERIAL**

**Elevated lipoprotein(a) and lipoprotein-associated phospholipase A_2_ are associated with unfavorable functional outcomes in patients with ischemic stroke**

**Table S1. Comparisons of Baseline characteristics between included patients and excluded patients**

| **Characteristics** | **All**  **(n=10491)** | **Included patients (n=9709)** | **Excluded patients (n=782)** | ***p*-value** |
| --- | --- | --- | --- | --- |
| **Demographic characteristics** |  |  |  |  |
| Age, years, median (IQR), y  Male, n (%) | 63.0 (54.0, 70.0)  7237 (69.0) | 63.0 (55.0, 70.0)  6689 (68.9) | 61.5 (53.0, 70.0)  548 (70.1) | 0.03  0.49 |
| BMI (kg/m^2^), median (IQR)  Current smoker, n (%) | 24.5 (22.6, 26.6)  3377 (32.2) | 24.5 (22.6, 26.6)  3102 (31.9) | 24.2 (22.5, 26.6)  275 (35.2) | 0.25  0.06 |
| **Medical history, n (%)** |  |  |  |  |
| Hypertension | 6612 (63.0) | 6112 (63.0) | 500 (63.9) | 0.58 |
| Diabetes mellitus  Hyperlipidemia  **TIA, n (%)** | 2532 (24.1)  844 (8.0)  238 (2.3) | 2340 (24.1)  778 (8.0)  221 (2.3) | 192 (24.5)  66 (8.4)  17 (2.2) | 0.78  0.67  0.85 |
| **TOAST subtype, n (%)** |  |  |  | 0.91 |
| LAA  CE  SAO  Others | 2685 (25.6)  690 (6.6)  2330 (22.2)  4786 (45.6) | 2488 (25.6)  639 (6.6)  2162 (22.3)  4420 (45.5) | 197 (25.2)  51 (6.5)  168 (21.5)  366 (46.8) |  |
| **TPA, n (%)**  **Yes**  **No** | 984 (9.4)  9507 (90.6) | 908 (9.3)  8801 (90.6) | 76 (9.7)  706 (90.3) | 0.73 |
| **Laboratory test**  SBP at admission (mmHg), median (IQR) | 149.0 (135.0, 165.0) | 149.0 (135.0, 165.0) | 148.2 (136.5, 164.5) | 0.82 |
| FPG (mM), median (IQR) | 5.6 (4.9, 7.0) | 5.6 (4.9, 7.0) | 5.6 (4.9, 7.1) | 0.85 |
| Baseline LDL-C (mM), median (IQR)  Baseline HDL-C (mM), median (IQR)  Baseline TG (mM), median (IQR)  Baseline hsCRP (mg/L), median (IQR)  Baseline Lp-PLA_2_ (ng/mL), median (IQR)  **Discharge Medication, n (%)**  Lipid-lowering drugs  Antiplatelet agents | 2.3 (1.7, 3.0)  1.1 (0.9, 1.3)  1.4 (1.0, 1.9)  1.8 (0.8, 4.8)  175.0 (126.6, 226.1)  9721 (92.9)  9599 (91.8) | 2.3 (1.8, 3.0)  1.1 (0.9, 1.3)  1.4 (1.0, 1.9)  1.8 (0.8, 4.8)  175.6 (127.3, 226.4)  9040 (93.4)  8925 (92.2) | 2.3 (1.7, 2.9)  1.1 (0.9, 1.3)  1.4 (1.0, 1.9)  1.9 (0.8, 5.5)  163.2 (115.1, 219.8)  681 (87.4)  674 (86.5) | 0.12  0.65  0.13  0.89  0.0008  <0.0001  <0.0001 |

Continuous data are presented as median (interquartile range, IQR), and categorical variables are presented as %.

Abbreviations: BMI: body mass index; LAA: large-artery atherosclerosis; CE: cardioembolism; SAO: small artery occlusion; TIA: transient ischemic attack; TPA: tissue plasminogen activator; SBP: systolic blood pressure; FPG: fasting plasma glucose; LDL-C: low-density lipoprotein cholesterol; HDL-C: high-density lipoprotein cholesterol; TG: triglyceride; hsCRP: high-sensitivity C-reactive protein; Lp-PLA_2_: lipoprotein-associated phospholipase A_2_.

**Table S2. Association between the levels of Lp(a) and functional outcomes at 3 months after excluding** stroke recurrence

| Event rate | | OR (95% confidence interval) | |
| --- | --- | --- | --- |
|  |  | Unadjusted | Adjusted |
| **mRS**≥**3 at 3 months**  Lp(a)(Q1)  Lp(a)(Q2)  Lp(a)(Q3)  Lp(a)(Q4) | 9.61  12.10  12.05  14.72 | Reference  1.29 (1.07-1.56)  1.29 (1.07-1.56)  1.62 (1.35-1.95) | Reference  1.26 (1.02-1.56)  1.20 (0.97-1.48)  1.37 (1.11-1.69) |

Adjust for age, sex, BMI, Diabetes mellitus, LDL-C, HDL-C, TG, Lp-PLA_2_, TOAST subtype and NIHSS score at admission.

Abbreviations: BMI: body mass index; LDL-C: low-density lipoprotein cholesterol; HDL-C: high-density lipoprotein cholesterol; TG: triglyceride; Lp-PLA_2_: lipoprotein-associated phospholipase A_2_; NIHSS: National Institutes of Health Stroke Scale.

**Table S3. Association between the Lp-PLA_2_ and functional outcomes at 3 months and 1 year**

|  | Event rate | OR (95% confidence interval) | |
| --- | --- | --- | --- |
|  |  | Unadjusted | Adjusted |
| **mRS≥3 at 3 months**  Lp-PLA_2_(Q1)  Lp-PLA_2_(Q2)  Lp-PLA_2_(Q3)  Lp-PLA_2_(Q4)  **mRS≥3 at 1 year**  Lp-PLA_2_(Q1)  Lp-PLA_2_(Q2)  Lp-PLA_2_(Q3)  Lp-PLA_2_(Q4) | 14.46  12.86  14.42  16.06  13.25  12.98  14.00  14.62 | Reference  0.87 (0.74-1.03)  1.00 (0.85-1.17)  1.13 (0.97-1.32)  Reference  0.98 (0.82-1.15)  1.07 (0.90-1.26)  1.12 (0.95-1.32) | Reference  0.84 (0.70-1.00)  0.92 (0.77-1.10)  1.00 (0.83-1.21)  Reference  0.97 (0.81-1.17)  1.06 (0.88-1.28)  1.09 (0.89-1.32) |

Adjust for age, sex, BMI, Diabetes mellitus, LDL-C, HDL-C, TG, TOAST subtype and NIHSS score at admission.

Abbreviations: BMI: body mass index; LDL-C: low-density lipoprotein cholesterol; HDL-C: high-density lipoprotein cholesterol; TG: triglyceride; NIHSS: National Institutes of Health Stroke Scale

**Table S4. Association between the levels of Lp(a) and functional outcomes at 3 months and 1 year**

|  | OR (95% confidence interval) | |
| --- | --- | --- |
|  | Unadjusted | Adjusted |
| **mRS≥3 at 3 months**  Lp(a)(Q1)  Lp(a)(Q2)  Lp(a)(Q3)  Lp(a)(Q4)  **mRS≥3 at 1 year**  Lp(a)(Q1)  Lp(a)(Q2)  Lp(a)(Q3)  Lp(a)(Q4) | Reference  1.16 (1.05-1.29)  1.19 (1.07-1.32)  1.32 (1.19-1.46)  Reference  1.12 (1.01-1.24)  1.15 (1.04-1.28)  1.23 (1.11-1.37) | Reference  1.13 (1.02-1.26)  1.12 (1.01-1.25)  1.17 (1.05-1.31)  Reference  1.06 (0.95-1.18)  1.08 (0.97-1.20)  1.10 (0.99-1.23) |

Adjust for age, sex, BMI, Diabetes mellitus, LDL-C, HDL-C, TG, Lp-PLA_2_, TOAST subtype and NIHSS score at admission.

Abbreviations: BMI: body mass index; LDL-C: low-density lipoprotein cholesterol; HDL-C: high-density lipoprotein cholesterol; TG: triglyceride; Lp-PLA_2_: lipoprotein-associated phospholipase A_2_; NIHSS: National Institutes of Health Stroke Scale

**Table S5. Comparisons of Baseline characteristics between included patients and excluded patients**

| **Characteristics** | **All**  **(n=14146)** | **Included patients (n=9709)** | **Excluded patients (n=4437)** | ***p*-value** |
| --- | --- | --- | --- | --- |
| **Demographic characteristics** |  |  |  |  |
| Age, years, median (IQR), y  Male, n (%) | 63.0 (54.0, 70.0)  9720 (68.7) | 63.0 (55.0, 70.0)  6689 (68.9) | 62.0 (54.0, 70.0)  3031 (68.3) | 0.11  0.49 |
| BMI (kg/m^2^), median (IQR)  Current smoker, n (%) | 24.5 (22.6, 26.5)  4503 (31.8) | 24.5 (22.6, 26.6)  3102 (31.9) | 24.5 (22.6, 26.5)  1401 (31.6) | 0.47  0.65 |
| **Medical history, n (%)** |  |  |  |  |
| Hypertension | 8887 (62.8) | 6112 (63.0) | 2775 (62.5) | 0.64 |
| Diabetes mellitus  Hyperlipidemia  TIA, n (%) | 3310 (23.4)  1075 (7.6)  306 (2.2) | 2340 (24.1)  778 (8.0)  221 (2.3) | 970 (21.9)  297 (6.7)  85 (1.9) | 0.004  0.006  0.17 |
| **TOAST subtype, n (%)** |  |  |  | 0.06 |
| LAA  CE  SAO  Others | 3667 (25.9)  881 (6.2)  3137 (22.2)  6461 (45.7) | 2488 (25.6)  639 (6.6)  2162 (22.3)  4420 (45.5) | 1179 (26.6)  242 (5.4)  975 (22.0)  2041 (46.0) |  |
| **TPA, n (%)**  Yes  No | 1257 (8.9)  12889 (91.1) | 908 (9.3)  8801 (90.6) | 349 (7.9)  4088 (92.1) | 0.004 |
| **Discharge Medication, n (%)**  Lipid-lowering drugs  Antiplatelet agents | 12947 (91.8)  12814 (90.8) | 9040 (93.4)  8925 (92.2) | 3907 (88.2)  3889 (87.8) | <0.0001  <0.0001 |

Continuous data are presented as median (interquartile range, IQR), and categorical variables are presented as %.

Abbreviations: BMI: body mass index; LAA: large-artery atherosclerosis; SAO: small artery occlusion; CE: cardioembolism; TIA: transient ischemic attack; TPA: tissue plasminogen activator; SBP: systolic blood pressure; FPG: fasting plasma glucose; LDL-C: low-density lipoprotein cholesterol; HDL-C: high-density lipoprotein cholesterol; TG: triglyceride; hsCRP: high-sensitivity C-reactive protein; Lp-PLA_2_: lipoprotein-associated phospholipase A_2_.

Table S6. Association between the levels of Lp(a) and functional outcomes at 3 months and 1 year

|  | OR (95% confidence interval) | |
| --- | --- | --- |
|  | Unadjusted | Adjusted |
| **mRS≥3 at 3 months**  Lp(a)(Q1)  Lp(a)(Q2)  Lp(a)(Q3)  Lp(a)(Q4)  **mRS≥3 at 1 year**  Lp(a)(Q1)  Lp(a)(Q2)  Lp(a)(Q3)  Lp(a)(Q4) | Reference  1.21 (1.05-1.40)  1.31 (1.14-1.51)  1.58 (1.38-1.81)  Reference  1.16 (1.00-1.34)  1.25 (1.08-1.44)  1.47 (1.27-1.69) | Reference  1.17 (1.00-1.37)  1.22 (1.05-1.43)  1.34 (1.14-1.56)  Reference  1.09 (0.92-1.27)  1.16 (0.99-1.36)  1.26 (1.07-1.47) |

Adjust for age, sex, BMI, Diabetes mellitus, LDL-C, HDL-C, TG, Lp-PLA_2_, TOAST subtype and NIHSS score at admission.

Abbreviations: BMI: body mass index; LDL-C: low-density lipoprotein cholesterol; HDL-C: high-density lipoprotein cholesterol; TG: triglyceride; Lp-PLA_2_: lipoprotein-associated phospholipase A_2_; NIHSS: National Institutes of Health Stroke Scale

**Table S7. Association of functional outcomes grouped by different levels of Lp(a) and Lp-PLA_2_**

|  | OR (95% confidence interval) | |
| --- | --- | --- |
|  | Unadjusted | Adjusted |
| **mRS≥3 at 3 months**  Lp(a)<median and Lp-PLA_2_<median  Lp(a)<median and Lp-PLA_2_≥median  Lp(a)≥median and Lp-PLA_2_<median  Lp(a)≥median and Lp-PLA_2_≥median  **mRS≥3 at 1 year**  Lp(a)<median and Lp-PLA_2_<median  Lp(a)<median and Lp-PLA_2_≥median  Lp(a)≥median and Lp-PLA_2_<median  Lp(a)≥median and Lp-PLA_2_≥median | Reference  0.99 (0.85-1.14)  1.18 (1.03-1.35)  1.41 (1.24-1.60)  Reference  1.05 (0.91-1.22)  1.23 (1.07-1.41)  1.34 (1.17-1.53) | Reference  0.93 (0.79-1.10)  1.08 (0.93-1.26)  1.20 (1.03-1.40)  Reference  1.06 (0.90-1.25)  1.15 (0.99-1.34)  1.24 (1.05-1.45) |

Adjust for age, sex, BMI, Diabetes mellitus, LDL-C, HDL-C, TG, TOAST subtype and NIHSS score at admission.

Abbreviations: BMI: body mass index; LDL-C: low-density lipoprotein cholesterol; HDL-C: high-density lipoprotein cholesterol; TG: triglyceride; NIHSS: National Institutes of Health Stroke Scale.

**Supplemental Figures and Figure legends**


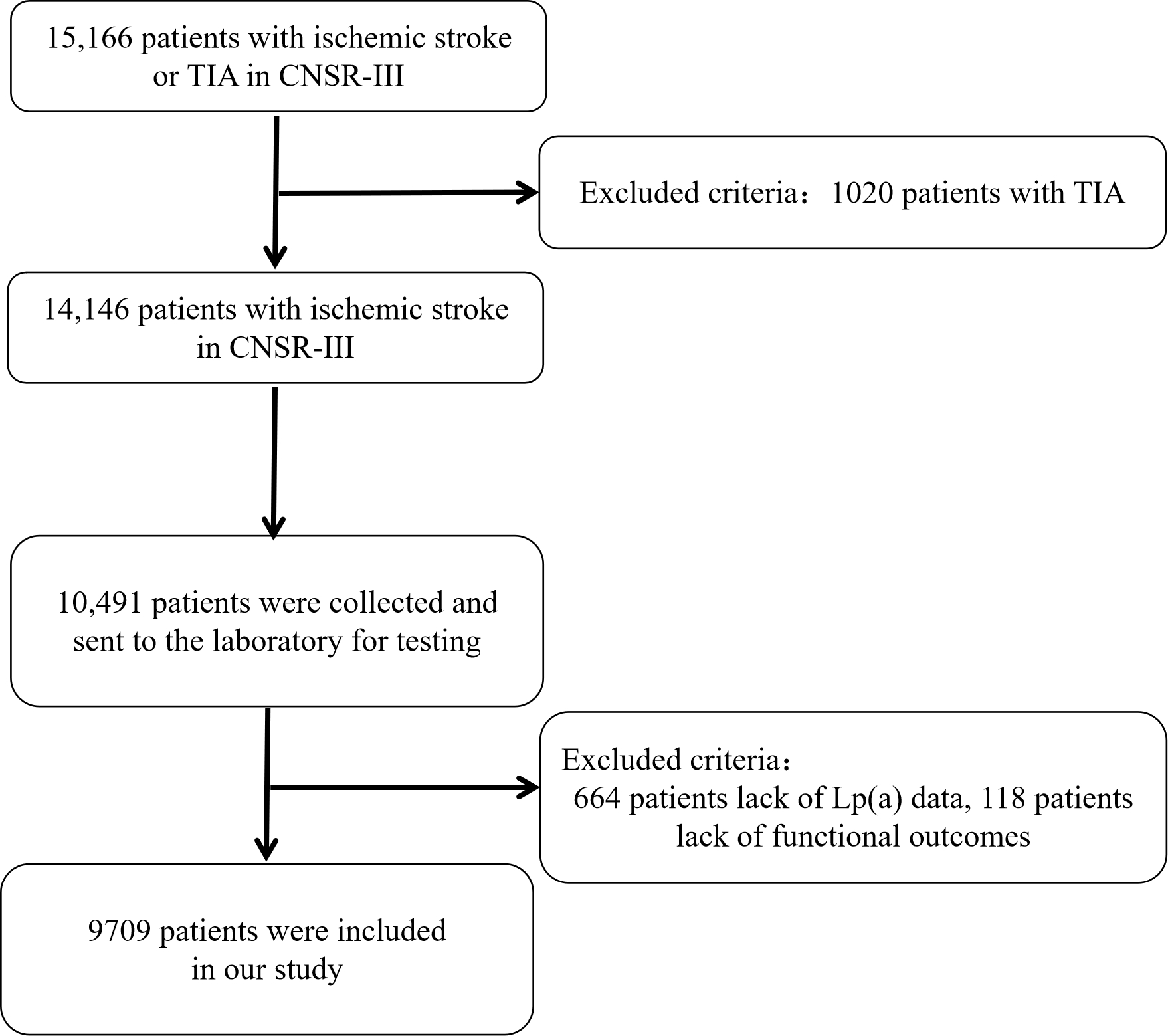


Figure S1. Flowchart of patients’ inclusion analysis strategy

**

**

Figure S2. The association between the levels of Lp(a) and outcomes at 3 months


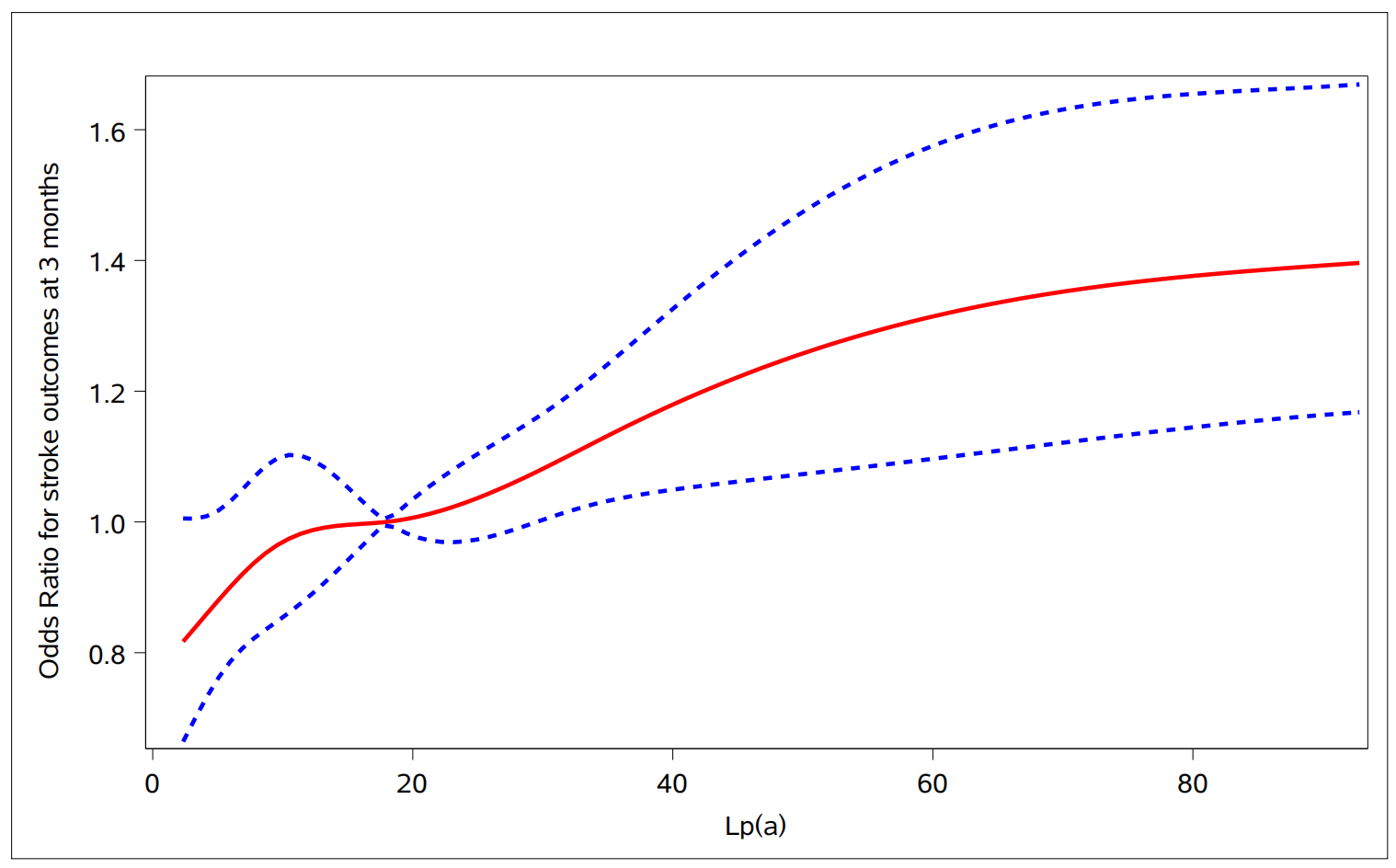

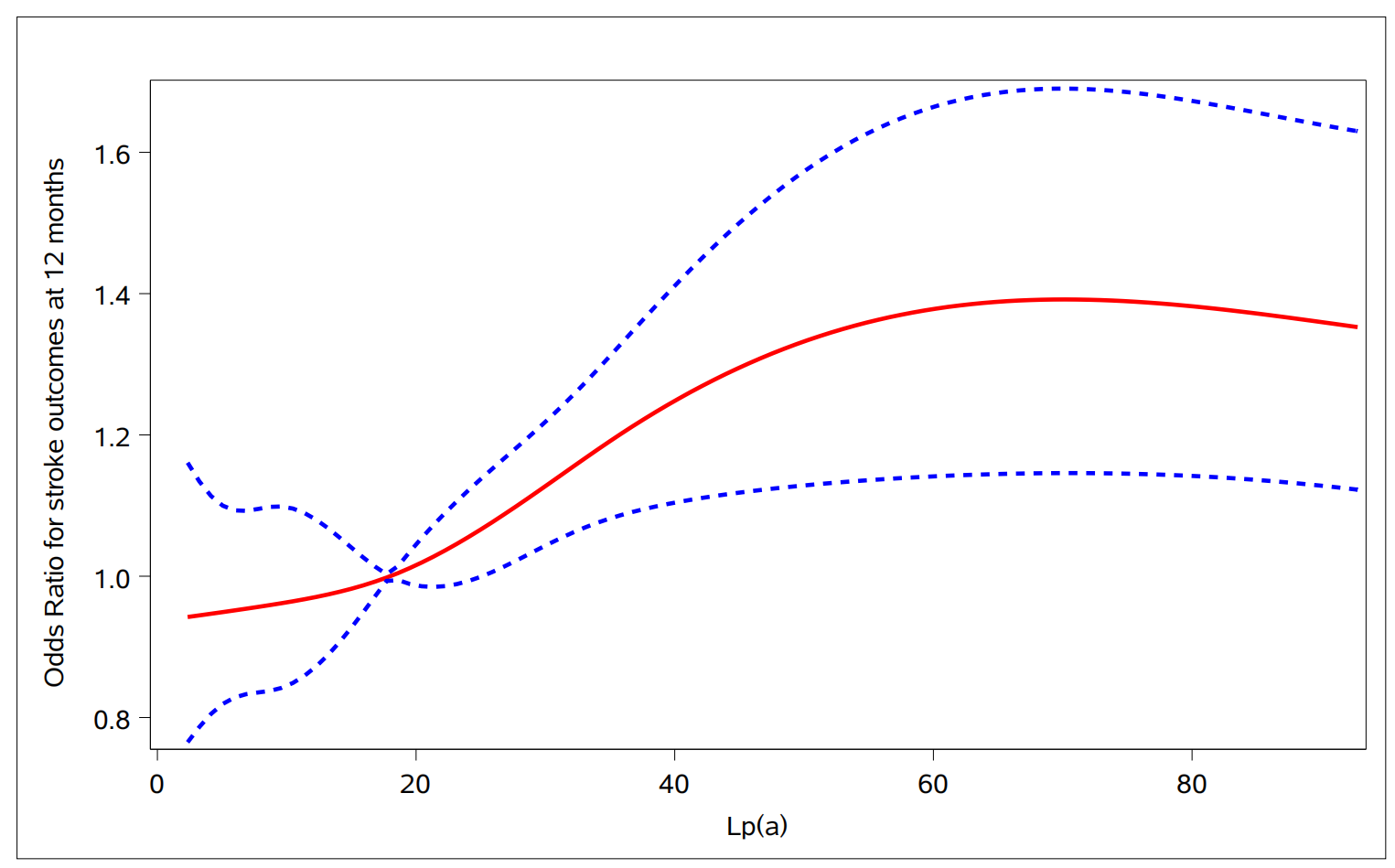


Figure S3. The association of Lp(a) and functional outcomes in patients with ischemic stroke at 3 months and 1 year
